# Descriptive Epidemiology of Acute Febrile Illness Patients in Nigeria: Pathogens of Global Public Health Significance Detected Using a Multi-Pathogen Detection Tool

**DOI:** 10.1093/cid/ciaf500

**Published:** 2025-11-20

**Authors:** Lauren P Courtney, Vivian Kwaghe, Cyril Erameh, Jay Osi Samuels, Claire A Quiner, Jean H Kim, Osahogie Isaac Edeawe, Nankpah Godsave Vongdip, Adamu Zigwai Ephraim, Onyia Justus Ejike, Ikponmwosa Odia, Kat Asman, Philippe Chebu, Jacqueline Agbukor, Oladimeji Damilare Matthew, Victoria Orok, Femi Owolagba, Blessed Okhiria, Ephraim Ogbaini-Emovon, Walter Mary Odion, Blessing Amierhobhiye Obagho, Richard Fayomade, Reuben Eifediyi, Joseph Okoeguale, Julius Imoyera, Emmanuel A Oga

**Affiliations:** Solutions, RTI International, Durham, North Carolina, USA; Internal Medicine, University of Abuja Teaching Hospital, Gwagwalada, Federal Capital Territory, Nigeria; Institute of Viral and Emergent Pathogens Control and Research, Irrua Specialist Teaching Hospital, Edo, Nigeria; Laboratory Services, APIN Public Health Initiatives, Federal Capital Territory, Nigeria; Solutions, RTI International, Durham, North Carolina, USA; Solutions, RTI International, Durham, North Carolina, USA; Institute of Viral and Emergent Pathogens Control and Research, Irrua Specialist Teaching Hospital, Edo, Nigeria; Internal Medicine, University of Abuja Teaching Hospital, Gwagwalada, Federal Capital Territory, Nigeria; Solutions, RTI International, Durham, North Carolina, USA; Internal Medicine, University of Abuja Teaching Hospital, Gwagwalada, Federal Capital Territory, Nigeria; Institute of Viral and Emergent Pathogens Control and Research, Irrua Specialist Teaching Hospital, Edo, Nigeria; Solutions, RTI International, Durham, North Carolina, USA; Laboratory Services, APIN Public Health Initiatives, Federal Capital Territory, Nigeria; Institute of Viral and Emergent Pathogens Control and Research, Irrua Specialist Teaching Hospital, Edo, Nigeria; Internal Medicine, University of Abuja Teaching Hospital, Gwagwalada, Federal Capital Territory, Nigeria; Internal Medicine, University of Abuja Teaching Hospital, Gwagwalada, Federal Capital Territory, Nigeria; Laboratory Services, APIN Public Health Initiatives, Federal Capital Territory, Nigeria; Internal Medicine, University of Abuja Teaching Hospital, Gwagwalada, Federal Capital Territory, Nigeria; Institute of Viral and Emergent Pathogens Control and Research, Irrua Specialist Teaching Hospital, Edo, Nigeria; Institute of Viral and Emergent Pathogens Control and Research, Irrua Specialist Teaching Hospital, Edo, Nigeria; Institute of Viral and Emergent Pathogens Control and Research, Irrua Specialist Teaching Hospital, Edo, Nigeria; Laboratory Services, APIN Public Health Initiatives, Federal Capital Territory, Nigeria; Institute of Viral and Emergent Pathogens Control and Research, Irrua Specialist Teaching Hospital, Edo, Nigeria; Institute of Viral and Emergent Pathogens Control and Research, Irrua Specialist Teaching Hospital, Edo, Nigeria; Institute of Viral and Emergent Pathogens Control and Research, Irrua Specialist Teaching Hospital, Edo, Nigeria; Solutions, RTI International, Durham, North Carolina, USA; ClineEpi Partners, Columbia, Maryland, USA

**Keywords:** acute febrile illness, AFI surveillance, infectious disease surveillance, multi-pathogen detection

## Abstract

**Background:**

Undifferentiated acute febrile illness (AFI) is a diagnosis that elicits public health concern, as it may be the result of an undiagnosed case of a pathogen of epidemic potential. This study compares the hospital-based incidence and distribution of AFI-causing pathogens in Nigeria, utilizing polymerase chain reaction (PCR)-based surveillance.

**Methods:**

Patients presenting with AFI at tertiary hospitals in Federal Capital Territory (north-central) and Edo (south) were enrolled from August 2023 to September 2024. Patients were screened for 25 pathogens using the TaqMan Array Card multi-pathogen PCR. The analysis compared the occurrence and distribution of pathogens, alongside environmental and demographic factors.

**Results:**

One thousand two hundred febrile patients were enrolled, 600 (50%) from each site with 37.5% children. Twenty pathogens were detected in 694 (57.8%) enrollees. The three most commonly detected pathogens in this AFI population were *Rickettsia* spp. (n = 312, 26.0%), *Plasmodium* spp. (n = 293, 24.4%), and Lassa fever virus (n = 184. 15.3%). Other pathogens include *Brucella* spp. (n = 14), *Neisseria meningitidis* (n = 12), dengue virus (n = 10), o’nyong’nyong virus (n = 7), chikungunya virus (n = 6), Crimean-Congo hemorrhagic fever virus (n = 4), and 11 additional pathogens. No statistically significant differences were shown between the regions.

**Conclusions:**

This epidemiologic study screening for the presence of 25 pathogens was instrumental in identifying pathogens of public health significance, including the first detection of four pathogens in humans in Nigeria. These findings suggest that there are likely other undetected cases of these pathogens, highlighting the need for continued surveillance to monitor and respond to emerging health threats.

## Teaser

The surveillance of acute febrile illness etiologies in Nigeria (SAFIAN) study used multi-pathogen Taqman array cards, detecting twenty pathogens including *Rickettsia*, *Plasmodium,* Lassa fever virus and other pathogens of public health significance, highlighting the need for ongoing infectious disease surveillance.

Acute febrile illness (AFI) is a clinical syndrome characterized by the sudden onset of fever and other non-specific symptoms, with potential for creating significant public health challenges worldwide [1]. Because AFI is a common non-specific manifestation of a wide range of infectious diseases, spanning viral, bacterial, and protozoan etiologies, it can become a significant public health challenge in healthcare systems where diagnostic capacity is limited. From a clinical standpoint, accurate and timely diagnosis of the cause of AFI is critical for effective patient management. In malaria endemic areas, misdiagnosis of AFI cases as malaria is common due to its non-specific symptoms, which often mimic other febrile illnesses [2, 3]. The presence of malaria parasites may not indicate active disease, leading to potential misdiagnosis and inappropriate treatment [4–7]. In endemic areas, individuals may carry the parasite without exhibiting severe symptoms, complicating accurate diagnosis, and overlooking additional coinfections. Overdiagnosis of malaria results in missed opportunities to identify and treat alternative, and often life-threatening, infections such as bacterial sepsis, typhoid fever, and arboviral diseases [7].

From an epidemiological standpoint, accurate AFI diagnosis is the cornerstone of effective public health surveillance. The ability to detect and characterize the etiological agents of AFI has far-reaching implications for public health planning, outbreak preparedness, and the allocation of resources. The absence of comprehensive, laboratory-based AFI surveillance systems in many low- and middle-income countries has created significant blind spots in understanding the burden and distribution of infectious diseases.

Understanding the epidemiology of AFI is crucial, yet global data gaps hinder effective surveillance and response [8]. The scarcity of comprehensive data on AFI limits our ability to conduct thorough surveillance, making it difficult to identify and address the diverse etiologies. This lack of information impedes the development of targeted interventions and effective public health strategies. Clinicians are often challenged by a lack of laboratory diagnostics, but assigning a definitive AFI cause based solely on symptoms is particularly challenging due to the overlapping clinical presentations of various infectious diseases [9, 10], caused by a wide range of viral, bacterial, and protozoan pathogens [11]. Understanding the pathogens present in a population is crucial for conducting appropriate laboratory diagnostics and ensuring effective treatment, as demonstrated by the identification of Crimean-Congo hemorrhagic fever cases during the Sudan virus disease outbreak in Uganda [12]. Enhanced surveillance and research are essential to improve diagnostic accuracy and public health outcomes [13].

Epidemiological patterns and trends are driven by ecological, climatic, and socioeconomic factors. The highly populated country of Nigeria is a predominantly tropical country in West Africa, characterized by diverse geography and climates, ranging from arid Sahelian savanna in the north to humid tropical rainforests in the south. Nigeria's diverse landscape creates diversity in disease between the south and the central or northern areas. The humid tropical climate and extensive wetlands of the southern regions create unique environments for vector-borne diseases with high endemicity, including malaria [14]. These conditions are distinct from the northern region's environment, which is characterized by a semi-arid climate, with epidemiological trends that indicate a higher incidence of malaria transmission than the southern part of the country [14].

The aim of this paper is to describe the demographics and behaviors among acutely febrile populations in two distinct regions of Nigeria and the common clinical practices for acute febrile treatment in these populations. We present the screening results for 25 pathogens of global public health importance, using a molecular, multi-pathogen screening tool and provide the first publicly available report for detection of 4 pathogens in humans in Nigeria, with the first molecular detection of an additional seven pathogens.

## METHODS

We conducted a multisite study, enrolling 450 children and 750 adults who were seeking medical care at two hospitals in Nigeria; the University of Abuja Teaching Hospital (UATH) in Gwagwalada, Federal Capital Territory (FCT), and Irrua Specialist Teaching Hospital in Irrua, Edo State. Participants were enrolled over a 12-month period at each site, from August 2023 to August 2024 in FCT and from September 2023 to September 2024 in Edo.

Patients identified with fever were recruited from multiple hospital departments to ensure representation. FCT recruited from the following units: General Outpatient Department, Gynae Emergency Unit, Medical Outpatient Department, Medical Outpatient Department, Emergency Pediatric Unit, and Pediatrics Outpatient. Edo recruited from the following: Accident and Emergency, General Outpatient clinic, Consultant Outpatient clinic, Emergency Pediatric Unit, Obstetrics and Gynecology, and Pediatric Outpatient. Study coordinators implemented a sampling framework of 37.5% children each month to attain >99% power when comparing malaria in children and adults; additionally, they aimed to recruit 50 participants per site each month to capture seasonal effects within the study population.

Potential participants were approached by study coordinators who described the Surveillance of Acute Febrile illness Aetiologies in Nigeria (SAFIAN) study, their requested role, and the monetary incentive (4000 Naira, ∼$5USD) for their participation. Patients who agreed were screened for the following criteria.

Inclusion Criteria included patients with:

- Documented measured axillary, rectal, tympanic, or oral temperature ≥37.5°C, with fever onset within the last 10 days,
- History of self-reported fever with onset within the last 10 days that persisted for 2–7 days, and
- Age ≥5 years.

Exclusion Criteria included patients:

- Returning for continued treatment of a fever of known cause,
- Who were already enrolled in SAFIAN,
- Presenting with an obvious cause of fever (eg, urinary tract infection, open wounds),
- Who do not provide consent/assent to participate,
- Who cannot provide sufficient blood draw.

After screening potential participants for inclusion, study coordinators obtained informed consent from eligible and willing patients and enrolled them into SAFIAN; assent was obtained for minors along with parental consent. Next, the study coordinator administered the Patient Survey, asking enrollees demographic and risk factor questions including exposure to animals, occupation and activities with known risk for pathogen exposure, eg, slaughtering animals, and use of bed nets. Specimens were collected from each enrollee by a trained phlebotomist: a 6 mL whole blood sample (3 mL in an EDTA vacutainer tube and 3 mL in a serum vacutainer tube). For patients who presented with characteristic poxvirus lesions, domed and cratered in appearance, a swab was also collected from the lesion using standard procedures [15, 16]. Blood samples were transferred to on-site hospital laboratories for processing and analysis. Serum samples were stored at ≤ −70°C. Total nucleic acid (TNA) extractions were conducted on whole blood and pox samples. The remaining whole blood samples and purified TNA extracts were stored at ≤ −70°C. Thermo Fisher's TaqMan Array Card (TAC) platform was used to screen all patient TNA specimens [17]. The TAC results were reviewed for quality and accuracy. Serologic tests were conducted on samples at FCT using Vircell (Granada, Spain) and Innovative Diagnostics (Grabels, France) following manufactures' protocols. See Supplementary material for additional details.

TAC can be customized for simultaneous detection of multiple pathogens; accordingly, we developed methods for researchers to prioritize the selection of pathogens for these multi-pathogen detection tools. First, a list of pathogens of global public health concern were assessed for their transmission potential in Nigeria, as defined by environmental requirements, habitat suitability for the reservoir and vector, and previous detection in humans and animals in Nigeria [18]. Next, we developed a model for pathogen selection for surveillance studies. Steps in this model included defining the study objectives, evaluating transmission potential of selected pathogens, and defining the pathogen inclusion/exclusion criteria based on study aims [19]. Using these frameworks, we selected 25 molecular targets for inclusion on the TAC panel (see Table 1).

**Table 1.** List of Molecular Targets Included on Customized SAFIAN Thermo Fisher's TaqMan Array Cards

Disease	Target	Abbreviation	Notes
Pathogen type			
Bacteria			
Bartonellosis	*Bartonella* spp.	BART	*…*
Brucellosis	*Brucella* spp.	BRUC	*…*
Q fever	*Coxiella burnetii*	CBUR	*…*
Leptospirosis	*Leptospira* spp.	LEPT	*…*
Meningococcal disease	*Neisseria meningitidis*	NMEN	Included as a singlet, as detection was unlikely.
Rickettsial disease	*Rickettsia* spp.	RICK	*…*
Salmonellosis	Pan-Salmonella	pSALM	…
Plague	*Yersinia pestis*	YPES	*…*
Protozoan	…	…	…
Leishmaniasis	*Leishmania* spp.	LEISH	…
Malaria	*Plasmodium* spp.	PLAS	Included as a singlet as this is commonly detected clinically and tested for by local physicians.
Trypanosomiasis	*Trypanosoma brucei*	TBRU	*…*
Viral	…	…	…
Crimean-Congo hemorrhagic fever	Crimean-Congo hemorrhagic fever virus	CCHFV	…
Chikungunya	Chikungunya virus	CHIKV	…
Dengue fever	Dengue virus	DENV	…
Hepatitis E	Hepatitis E virus	HEV	…
Hantavirus syndrome	Hantavirus	HTNV	…
Lassa fever	Lassa virus	LASV	Study results include those detected by TAC as well as those detected via *RealStar Lassa Virus RT-PCR*.
Marburg virus disease and Ebola virus disease	Marburg/Ebola virus	MARV/EBOV	Thermo Fisher custom designed the assay specifically for the SAFIAN study.
Mpox	Monkeypox virus	MPOX	Included as a singlet because the Pan-Orthopox wells provided duplication.
O'nyong'nyong fever	O'nyong'nyong virus	ONNV	…
Various diseases including Mpox, vaccinia, cowpox	Pan-Orthopox virus	pOPXV	Combined with the Mpox result, this result can also be used to identify non-Mpox Orthopox viruses. Included as a singlet because the MPOX wells provided duplication. Thermo Fisher custom designed the assay specifically for the SAFIAN study.
Rift Valley fever	Rift Valley fever virus	RVFV	…
West Nile	West Nile virus	WNV	…
Yellow fever	Yellow fever virus	YFV	…
Zika	Zika virus	ZIKV	…

Abbreviations: SAFIAN, Surveillance of Acute Febrile Infectious Aetiologies in Nigeria; TAC, TaqMan Array Cards; RT PCR, real time polymerase chain reaction.

In addition to TAC, Lassa virus testing was performed by Edo as standard clinical practice to diagnose a subset of participants using the RealStar Lassa Virus RT- polymerase chain reaction (PCR) kit (Altona Diagnostics; Hamburg, Germany) and following the hospital lab's standard operating procedures for testing. The study also performed Lassa virus PCR testing using the same kit for all samples from FCT and the remaining untested samples from Edo. The remaining Edo samples were tested using the same hospital procedures. For FCT, the testing procedures were modified to align with the thermocycler present at the site.

After patient discharge, the study coordinator extracted medical record data for inclusion in a REDCap data form including diagnosis, treatment, and the results of diagnostic tests.

To optimize data collection and quality, data were directly entered into REDCap forms by the study coordinators, using validation checks to ensure quality. All clinical data collected from the participant (Screening, Patient Survey, and Discharge Form), were entered directly into REDCap by the study coordinator. Barcodes and barcode stickers were used to link the patient's information, forms, specimens, and results.

Data were downloaded from the REDCap system and prepared for analyses accordingly. As this manuscript is descriptive in nature, we describe the participating population and the associated behaviors and potential risk factors for AFI. Characteristics were summarized using frequencies and percentages for categorical characteristics and means and standard deviations were used for continuous characteristics. Differences were looked at by location (FCT and Edo) and by age (youth and adult) to determine significance using chi-square or t-tests. Analyses were performed using SAS 9.4 (Cary, North Carolina).

## RESULTS

### Study Population

From 22 August 2023, to 4 September 2024, a total of 1217 patients were screened from two hospitals; 1200 eligible AFI patients consented and enrolled into the SAFIAN study, with 225 children (5–17 years) and 375 adults (18–108 years) from each site (Table 2). Slightly more female participants (51.0%) were enrolled, with significantly more men in Edo (55.2%) than in FCT (42.8%), and significantly more boys (54.4%) than adult men (45.7%). Sixty-four percent of adults were married. Two percent of women/girls were pregnant.

**Table 2.** Demographic Characteristics of Enrolled AFI Participants, by Site and Age

		Study Sites		Age	
Characteristic	Total (N = 1200)	FCT (n = 600)	Edo (n = 600)	Adult (n = 750)	Children (n = 450)
Age (mean [min, max])	…	…	…	…	…
Youth	11.5 (5.0–17.0)	11.0 (5.0–17.0)	12.0 (5.0–17.0)**	…	…
Adult	41.8 (18.0–108.0)	41.4 (18.0–90.0)	42.2 (18.0–108.0)	…	…
Age (N [%])	…	…	…	…	…
Adult	750 (62.5%)	375 (62.5%)	375 (62.5%)	…	…
Age (N [%])	…	…	…	…	…
5–12	254 (21.2%)	143 (23.8%)	111 (18.5%)	0 (0.0%)	254 (56.4%)
13–17	196 (16.3%)	82 (13.7%)	114 (19.0%)	0 (0.0%)	196 (43.6%)
18–49	533 (44.4%)	274 (45.7%)	259 (43.2%)	533 (71.1%)	0 (0.0%)
≥50	217 (18.1%)	101 (16.8%)	116 (19.3%)	217 (28.9%)	0 (0.0%)
Gender	…	…	…	…	…
Male	588 (49.0%)	257 (42.8%)	331 (55.2%)*	343 (45.7%)	245 (54.4%)
Female	612 (51.0%)	343 (57.2%)	269 (44.8%)	407 (54.3%)	205 (45.6%)
Pregnant	12 (2.0%)	5 (1.5%)	7 (2.6%)	11 (2.7%)	1 (0.5%)
Marital Status	…	…	…	…	…
Married	483 (40.3%)	234 (39.0%)	249 (41.5%)	481 (64.1%)*	2 (0.4%)
Occupation	…	…	…	…	…
Pupil/student	514 (42.8%)	244 (40.7%)	270 (45.0%)*	89 (11.9%)	425 (94.4%)
Trader/sales	151 (12.6%)	58 (9.7%)	93 (15.5%)	146 (19.4%)	5 (1.1%)
Civil servant/professional/technical/clerical	150 (12.5%)	86 (15.2%)	64 (10.7%)	150 (20%)	0 (0.0%)
Unemployed/retired/housewife	138 (11.6%)	79 (13.1%)	59 (9.9%)	99 (12.4%)	12 (2.7%)
Farmer	62 (5.2%)	14 (2.3%)	48 (8.0%)	60 (8.0%)	2 (0.4%)
Artisan	57 (4.8%)	35 (5.8%)	22 (3.7%)	53 (7.1%)	4 (0.9%)
Manual labor	37 (3.1%)	31 (5.1%)	6 (1.0%)	37 (4.8%)	1 (0.2%)
Healthcare worker	35 (2.9%)	26 (4.3%)	9 (1.5%)	35 (4.7%)	0 (0.0%)
Hunter/trader of game meat	5 (0.4%)	0 (0.0%)	5 (0.8%)	5 (0.7%)	0 (0.0%)
Butcher	4 (0.3%)	0 (0.0%)	4 (0.7%)	4 (0.5%)	0 (0.0%)
Mortician	1 (0.1%)	0 (0.0%)	1 (0.2%)	1 (0.1%)	0 (0.0%)
Other (teacher, police/military, transporter, religious/spiritual leader, miner)	46 (3.9%)	27 (4.5%)	19 (3.1%)	45 (5.9)	1 (0.2%)
Education level	…	…	…	…	…
No formal education	73 (6.1%)	15 (2.5%)	58 (9.7%)**	68 (9.1%)*	5 (1.1%)
Below primary	114 (9.5%)	93 (15.5%)	21 (3.5%)	2 (0.3%)	112 (24.9%)
Primary	177 (14.8%)	110 (18.3%)	67 (11.2%)	36 (4.8%)	141 (31.3%)
Below secondary	46 (3.8%)	12 (2.0%)	34 (5.7%)	37 (4.9%)	9 (2.0%)
Secondary	398 (33.2%)	179 (29.8%)	219 (36.5%)	249 (33.2%)	149 (33.1%)
Tertiary	346 (28.8%)	161 (26.8%)	185 (30.8%)	320 (42.7%)	26 (5.8%)
Other	36 (3.0%)	28 (4.7%)	8 (1.3%)	35 (4.7%)	1 (0.2%)
More than one education picked	10 (0.8%)	2 (0.3%)	8 (1.3%)	3 (0.4%)	7 (1.6%)
Season	…	…	…	…	…
Dry Season Start (15 Oct−14 Nov)	80 (6.7%)	39 (6.5%)	41 (6.8%)	53 (7.1%)	27 (6.0%)
Harmattan Period (15 Nov−14 Jan)	182 (15.2%)	86 (14.3%)	96 (16.0%)	140 (18.7%)	42 (9.3%)
Dry Season End (15 Jan−15 Mar)	303 (25.3%)	167 (27.8%)	136 (22.7%)	236 (31.5%)	67 (14.9%)
Wet Season (16 Mar−14 Oct)	635 (52.9%)	308 (51.3%)	327 (54.5%)	321 (42.8%)	314 (69.8%)*

*P < .0001, **P < .01.

Abbreviations: AFI, acute febrile illness; FCT, Federal Capital Territory.

The most common occupation was students, accounting for 94.4% of children and 11.9% of adults. Among adults, the most common occupations were students, traders, civil servants, farmers, and the unemployed. Edo's population included significantly more farmers (8%) and traders (15.2%), and the only participants reported to be hunters (0.8%) and butchers (0.7%), while FCT's population included more artisans (5.8%), healthcare workers (4.3%), and professional workers (3.9%).

Overall, 65% of participants received at least a secondary school education, with 31.8% completing a tertiary education. Significantly more participants in Edo (17.6%) had no formal education than FCT (4.2%). More Edo participants completed secondary school (68.7%) than FCT participants (61.3%).

**Figure 1.**
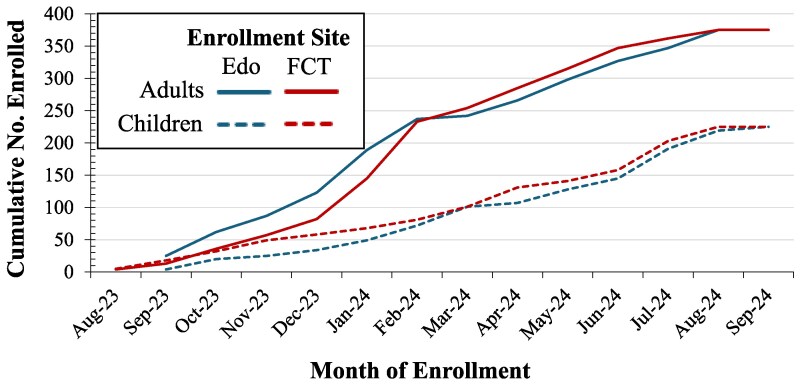
Cumulative SAFIAN enrollment numbers from August 2023 to September 2024, by age and site of recruitment. Blue lines indicates enrollment from Irrua Specialist Teaching Hospital in Edo state, and red lines indicates enrollment from the University of Abuja Teaching Hospital in the Federal Capital Territory (FCT). Solid lines indicates enrollment of adults and dotted lines are children (ages 5-18). Abbreviation: FCT, Federal Capital Territory.

**Figure 2.**
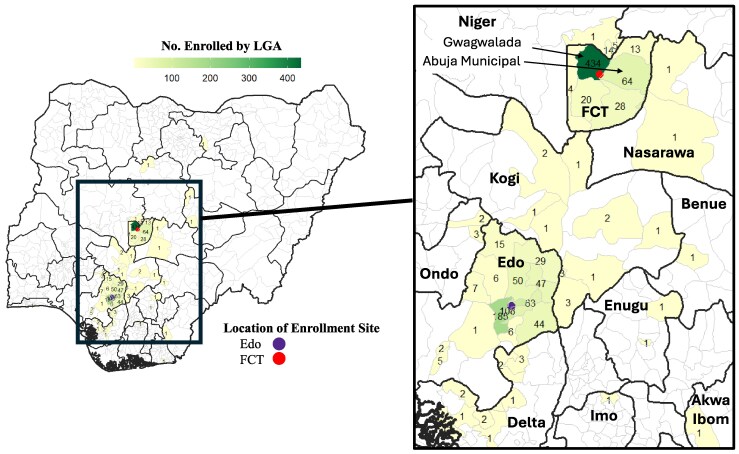
Numbers of SAFIAN enrollment, by LGA of patient residence. Black lines and bold text are state borders and names. Grey lines are LGA borders. White LGAs indicate that no one was enrolled from them. Abbreviations: SAFIAN, Surveillance of Acute Febrile Infectious Aetiologies in Nigeria; LGA, local government area; FCT, Federal Capital Territory.

Figure 1 shows recruitment distribution by age and month. Seasonal recruitment data are shown in Table 1, by age and site. A higher percentage of children (69.8%) were enrolled during the wet season (March–October) than adults during this period (42.8%) due to a focused recruitment strategy toward the end of recruitment to include more children. Figure 2 maps participant enrollment by place of residence.

Recruitment was evenly divided by site, with 94.7% of participants living in the same state where the hospital is located, 5.3% coming from other states.

### Participant Behaviors and Common Risk Factors

Reported bed net usage varied significantly between the two sites. Among participants enrolled in FCT, 47.3% (n = 284) reported bed net use at least once per week, as compared to only 18.3% (n = 110) among those recruited in Edo (Table 3). Additionally, 32% (n = 192) of the FCT-based population reported use 7 days per week, as compared to none in Edo. Use in children was significantly lower (30.0%) than in adults (34.5%).

**Table 3.** SAFIAN Participant Risk Behaviors, by Site and Age

			Study Sites		Age	
Characteristic		Total (N = 1200)	FCT (n = 600)	Edo (n = 600)	Adult (n = 750)	Children (n = 450)
Number of people in household (mean [min, max])		4.8 (1.0–25.0)	5.0 (1.0–25.0)	4.6 (1.0–13.0) ***	4.4 (1.0–20.0)	5.6 (1.0–25.0)*
Spent time in forest or bush past 14 d (%)		249 (20.8%)	48 (8.0%)	201 (33.5%)*	181 (24.1%)	68 (15.1%)**
Traveled outside of the country in the past month		3 (0.3%)	2 (0.3%)	1 (0.2%)	2 (0.3%)	1 (0.2%)
Bed net usage in the past 7 d		…	…	…	…	…
Any bed net use (%)		394 (32.8%)	284 (47.3%)	110 (18.3%)*	259 (34.5%)	135 (30.0%)
Bed net use: 0 d		806 (67.2%)	316 (52.7%)	490 (81.7%)*	491 (65.5%)	315 (70.0%)*
Bed net use: 1–6 d		202 (16.8%)	92 (15.3%)	110 (18.3%)	159 (21.2%)	43 (9.6%)
Bed net use: 7 d		192 (16.0%)	192 (32.0%)	0 (0.0%)	100 (13.3%)	92 (20.4%)
Insect bite exposure		…	…	…	…	…
Yes—bite in the past 14 d		630 (52.5%)	135 (22.5%)	495 (82.5%)*	395 (52.7%)	235 (52.2%)
No—bite in the past 14 d		443 (36.9%)	402 (67.0%)	41 (6.8%)	281 (37.5%)	162 (36.0%)
Don't know—bite in the past 14 d		127 (10.6%)	63 (10.5%)	64 (10.7%)	74 (9.9%)	53 (11.8%)
Mosquito bite in the past 14 d		602 (50.2%)	114 (19.0%)	488 (81.3%)*	379 (50.5%)	223 (49.6%)
Tick bite in the past 14 d		39 (3.3%)	0 (0.0%)	39 (6.5%)*	31 (4.1%)	8 (1.8%)
Other type of insect bite in the past 14 d		32 (2.7%)	17 (2.8%)	15 (2.5%)	18 (2.4%)	14 (3.1%)
Animal contact exposure the month before illness		…	…	…	…	…
Had contact with an animal		397 (33.1%)	250 (41.7%)	147 (24.5%)*	221 (29.5%)	176 (39.1%)**
Had contact with at least one alive animal		385 (32.1%)	250 (41.7%)	135 (22.5%)*	211 (28.1%)	174 (38.7%)**
Had contact with at least one dead animal		24 (2.0%)	0 (0.0%)	24 (4.0%)*	19 (2.5%)	5 (1.1%)
Had contact with at least one wildlife animal^a^		9 (0.8%)	1 (0.2%)	8 (1.3%)^	7 (0.9%)	2 (0.4%)
Animal contact type: Touch		381 (31.8%)	236 (39.3%)	145 (24.2%)*	209 (27.9%)	172 (38.2%)**
Animal contact type: Slaughter		30 (2.5%)	4 (0.7%)	26 (4.3%)*	23 (3.1%)	7 (1.6%)
Animal contact type: Delivery		11 (0.9%)	…	11 (1.8%)**	9 (1.2%)	2 (0.4%)
Animal contact type: Bite		3 (0.3%)	…	3 (0.5%)	1 (0.1%)	2 (0.4%)
Contact with living or dead/sick animals		…	…	…	…	…
Dog	*Any contact (dead or alive)*	198 (16.5%)	100 (16.7%)	98 (16.3%)	114 (15.2%)	84 (18.7%)
	*Alive*	197 (16.4%)	100 (16.7%)	97 (16.2%)	113 (15.1%)	84 (18.7%)
	*Dead/sick*	1 (0.1%)	(0%)	1 (0.2%)	1 (0.1%)	…
Cat	*Any contact*	51 (4.3%)	50 (8.3%)	1 (0.17%)*	25 (3.3%)	26 (5.8%)***
	*Alive*	51 (4.3%)	50 (8.3%)	1 (0.2%)*	25 (3.3%)	26 (5.8%)***
	*Dead/sick*	…	…	…	…	…
Cow	*Any contact*	18 (1.5%)	8 (1.3%)	10 (1.7%)	10 (1.3%)	3 (0.7%)
	*Alive*	13 (1.1%)	8 (1.3%)	5 (0.8%)	10 (1.3%)	3 (0.7%)
	*Dead/sick*	5 (0.4%)	…	5 (0.8%)	5 (0.7%)	…
Pig	*Any contact*	2 (0.2%)	2 (0.3%)	…	2 (0.3%)	…
	*Alive*	2 (0.2%)	2 (0.3%)	…	2 (0.3%)	…
	*Dead/sick*	…	…	…	…	…
Goat	*Any contact*	93 (7.75%)	59 (9.8%)	34 (5.7%)**	52 (6.9%)	41 (9.1%)
	*Alive*	89 (7.4%)	59 (9.8%)	30 (5%)	48(6.4%)	41 (9.1%)
	*Dead/sick*	4 (0.3%)	…	4 (0.7%)	4 (0.5%)	…
Sheep	*Any contact*	8 (0.7%)	8 (1.3%)	−**	7 (0.9%)	1 (0.2%)
	*Alive*	8 (0.7%)	8 (1.3%)	…	7 (0.9%)	1 (0.2%)
	*Dead/sick*	…	…	…	…	…
Chicken	*Any contact*	197 (16.5%)	154 (25.7%)	43 (7.2%)*	96 (12.8%)	101 (22.4%)*
	*Alive*	184 (15.3%)	154 (25.7%)	30 (5%)	87 (11.6%)	97 (21.6%)
	*Dead/sick*	13 (1.1%)	…	13 (2.2%)	9 (1.2%)	4 (0.9%)
Guinea fowl	*Any contact*	3 (0.25%)	3 (0.5%)	…	2 (0.3%)	1 (0.2%)
	*Alive*	3 (0.3%)	3 (0.5%)	…	2 (0.3%)	1 (0.2%)
	*Dead/sick*	…	…	…	…	…
Rodent	*Any contact*	8 (0.7%)	…	8 (1.3%)**	6 (0.8%)	2 (0.4%)
	*Alive*	4 (0.3%)	…	4 (0.7%)	4 (0.5%)	…
	*Dead/sick*	4 (0.3%)	…	4 (0.7%)	2 (0.3%)	2 (0.4%)
Monkey	*Any contact*	1 (0.1%)	1 (0.2%)	…	1 (0.1%)	…
	*Alive*	1 (0.1%)	1 (0.2%)	…	1 (0.1%)	…
	*Dead/sick*	…	…	…	…	…
Other animals	*Any contact*	4 (0.3%)	2 (0.3%)	2 (0.3%)	4 (0.5%)	…
	*Alive*	2 (0.2%)	2 (0.3%)	…	2 (0.3%)	…
	*Dead/sick*	2 (0.2%)	…	2 (0.3%)	2 (0.3%)	…
	…	…	…	…	…	…

*P < .0001, **P < .001, ***P < .01, ^ *P* < .05.

^a^Wildlife includes: rodent, monkey.

Abbreviations: SAFIAN, Surveillance of Acute Febrile Illness Aetiologies in Nigeria; FCT: Federal Capital Territory.

Contact with animals was higher among participants in FCT (41.7%) than those in Edo (24.5%). Overall, 31.8% of the population reported touching an animal and 3.7% of them reported slaughtering, delivering, or being bitten by an animal. The animals that enrollees most reported contact with were living dogs (16.4%), living chickens (15.3%), and living goats (7.4%). Contact with chickens was significantly more common among FCT enrollees (25.7%) as compared to Edo enrollees (5%). Contact with rodents was only reported by Edo enrollees (1.4%). Overall, children were nearly twice as likely to have contact with chickens (21.6%) as compared to adults (11.6%). Only 2.4% of enrollees reported having contact with a dead animal, which included rodents, chickens, goats, cattle, and a dog.

Only three participants reported traveling outside the country in the past month (to Benin Republic, Cameroon, and Niger).

### Pathogen Detection

The SAFIAN study detected 20 of the 25 pathogens screened (Table 4). At least one pathogen was detected in 57.8% (n = 694) of the 1200 enrollees. The three most commonly detected pathogens in this AFI population were *Rickettsia* spp. (n = 312), *Plasmodium* spp. (n = 293), and Lassa fever virus (n = 184). Distribution of *Rickettsia* spp. did not differ by site or age. *Plasmodium* spp. differed by age; 32.2% of children were *Plasmodium* spp. positive as compared to only 19.7% of adults. Lassa was detected primarily in Edo, with only three cases in FCT; Lassa virus results include a combination of TAC and single-pathogen PCR.

**Table 4.** Pathogens Detected for Enrolled AFI Participants, by Site and Age

Molecular Target	Total (N = 1200)	FCT (n = 600)	Edo (n = 600)	Adult (n = 750)	Children (n = 450)
Pathogens detected	*n* (%)	**…**	**…**	**…**	**…**
Rickettsial infections (*Rickettsia* spp.)	312 (26.0%)	161 (26.8%)	151 (25.2%)	184 (24.5%)	128 (28.4%)
Malaria (*Plasmodium* spp.)	293 (24.4%)	161 (26.8%)	132 (22.0%)	148 (19.7%)	145 (32.2%)
Lassa fever virus	187 (15.6%)	3 (0.5%)	184 (30.7%)	132 (17.6%)	55 (12.2%)
* Thermo Fisher TAC Lassa virus^a^*	*2* (*0.1%)*	*2* (*0.3%)*	…	*1* (*0.1%)*	*1 (0.2%)*
* RealStar Lassa Virus RT-PCR kit* *(LASV Lineages II- V)*	*185* (*15.4%)*	*1* (*0.2%)*	*184 (30.7%)*	*131* (*17.5%)*	*54 (12.0%)*
Brucellosis *(Brucella* spp*.)*	14 (1.2%)	2 (0.3%)	12 (2.0%)	9 (1.2%)	5 (1.1%)
Meningococcus (*Neisseria meningitidis*)	12 (1.0%)	6 (1.0%)	6 (1.0%)	10 (1.3%)	2 (0.4%)
Dengue virus	10 (0.8%)	5 (0.8%)	5 (0.8%)	8 (1.1%)	2 (0.4%)
O'nyong'nyong virus	7 (0.6%)	3 (0.5%)	4 (0.7%)	5 (0.7%)	2 (0.4%)
Chikungunya virus	6 (0.5%)	3 (0.5%)	3 (0.5%)	3 (0.4%)	3 (0.7%)
Crimean-Congo hemorrhagic fever virus	4 (0.3%)	2 (0.3%)	2 (0.3%)	3 (0.4%)	1 (0.2%)
Pan-Orthopoxvirus	2 (0.2%)	2 (0.3%)	…	2 (0.3%)	…
Zika virus	2 (0.2%)	1 (0.2%)	1 (0.2%)	2 (0.3%)	…
Bartonellosis *(Bartonella* spp*.)*	1 (0.1%)	1 (0.2%)	…	1 (0.1%)	…
Hepatitis E virus	1 (0.1%)	…	1 (0.2%)	…	1 (0.2%)
Monkeypox virus	1 (0.1%)	1 (0.2%)	…	1 (0.1%)	…
Rift Valley fever virus	1 (0.1%)	…	1 (0.2%)	1 (0.1%)	…
West Nile virus	1 (0.1%)	1 (0.2%)	…	…	1 (0.2%)
Q fever (*Coxiella burnetii*)	1 (0.1%)	…	1 (0.2%)	1 (0.1%)	…
Leptospirosis *(Leptospira* spp.*)*	1 (0.1%)	…	1 (0.2%)	1 (0.1%)	…
Plague *(Yersinia pestis*)	1 (0.1%)	1 (0.2%)	…	1 (0.1%)	…
Pan-Salmonella	1 (0.1%)	1 (0.2%)	…	1 (0.1%)	…
Total number of molecular targets detected	858	354	504	513	345
Pathogens not detected	…	…	…	…	…
Hantavirus	…	…	…	…	…
Leishmaniasis (*Leishmania* spp*.)*	…	…	…	…	…
Pan-filovirus (Ebola, Marburg, other*)*	…	…	…	…	…
Trypanosomiasis (*Trypanosoma brucei)*	…	…	…	…	…
Yellow fever virus	…	…	…	…	…

^a^TAC LASV assay may detect Lineage I since the 2 samples detected by TAC were not detected by the single-pathogen assay.

Abbreviations: FCT, Federal Capital Territory; LASV, Lassa virus; RT-PCR, Reverse transcription polymerase chain reaction; TAC, Taqman Array Card; AFI, Acute Febrile Illness.

Other pathogens detected were *Neisseria meningitidis* (n = 12), dengue virus (n = 10), Mpox (n = 1), and one case of the following: an Orthopoxvirus which was not Monkeypox, *Leptospira* spp., West Nile virus, or pan-Salmonella. No cases of Hantavirus, *Leishmania* spp., pan-filovirus, *Trypanosoma brucei*, or Yellow fever virus were detected.

Serological testing revealed evidence of prior or recent exposure to multiple pathogens, including *Rickettsia* spp. detected in 28.7% of patients, *Brucella* spp. in 3.9%, and Crimean-Congo hemorrhagic fever virus in 2.2% (Table 5). Given the prevalence of both *Rickettsia* spp. and *Plasmodium* spp. we conducted a comparative analysis of the group where only *Plasmodium* spp. was detected, only *Rickettsia* spp. was detected, or both were detected. There was no remarkable difference in the prevalence of any demographic, symptom, risk factor, or chronic health condition among these three groups (data not shown).

**Table 5.** Seropositivity Among Enrollees from FCT for Three Pathogens Detected Molecularly in SAFIAN

Immunological Target	Total (N = 596)
Crimean-Congo hemorrhagic fever virus	…
IgG and/or IgM positive	13 (2.2%)
*Rickettsia* spp.	…
IgG and/or IgM positive	171 (28.7%)
IgG positive	119 (20.0%)
IgM positive	66 (11.1%)
*Brucella* spp.	…
IgG and/or IgM positive	23 (3.9%)
IgG positive	8 (1.3%)
IgM positive	15 (2.5%)

Abbreviations: SAFIAN, Surveillance of Acute Febrile Infectious Aetiologies in Nigeria; FCT, Federal Capital Territory.

Coinfections were detected in 12.6% (n = 151) of participants, with detection of 2 (n = 143), 3 (n = 7) and 4 pathogens (n = 1). All coinfections had one or more of these three pathogens: *Plasmodium* spp., *Rickettsia* spp., or Lassa fever virus (Table 6).

**Table 6.** Frequency of Detection of Single and Multiple Pathogens by the SAFIAN Study, by Site and Age

Specimen Status	Total Number (%) Specimens	FCT	Edo	Adult	Child
With no pathogen detected	506 (42.2%)	308 (25.7%)	198 (16.5%)	339 (45.2%)	167 (37.1%)
At least one pathogen detected	694 (57.8%)	292 (24.3%)	402 (33.5%)	411 (54.8%)	283 (62.9%)
* One pathogen detected*	543 (45.3%)	232 (19.3%)	311 (25.9%)	320 (42.7%)	223 (49.6%)
* Codetection of 2 pathogens*	143 (11.9%)	59 (4.9%)	84 (7%)	84 (11.2%)	59 (13.1%)
* Codetection of 3 pathogens*	7 (0.6%)	1 (0.1%)	6 (0.5%)	6 (0.8%)	1 (0.2%)
* Codetection of 4 pathogens*	1 (0.1%)	…	1 (0.1%)	1 (0.1%)	…
Total specimens tested	1200 (100%)	600 (50%)	600 (50%)	750 (100%)	450 (100%)

Abbreviations: SAFIAN, Surveillance of Acute Febrile Infectious Aetiologies in Nigeria; FCT, Federal Capital Territory.

### Clinical Characteristics

Preexisting health conditions were reported in 28.3% of adults. In adults, the most common conditions were high blood pressure (18%), diabetes (6.4%), and HIV (2.5%). Diabetes was significantly higher in FCT than Edo.

Participants presented with a mean temperature of 38.1°C, including 7.1% with self-reported fever. Other symptoms included headache (66.9%), vomiting (33.3%), abdominal pain (23.6%), and cough (17.8%). Table 7 includes a full list of symptoms by site and by age.

**Table 7.** Hospital Diagnostics and Outcomes of Enrolled Febrile Participants, by Site and Age

		Study Sites		Age	
Characteristic	Total (N = 1200)	FCT (n = 600)	Edo (n = 600)	Adult (n = 750)	Children (n = 450)
Fever	…	…	…	…	…
Fever duration (mean [min, max])	2.8 (0.0–9.0)	2.7 (0.0–9.0)	2.9 (0.0–8.0)	2.9 (0.0–9.0)	2.7 (0.0–8.0)
Axillary temperature (°C)	…	…	…	…	…
Temperature (mean [min, max])	38.1 (35.6–41.1)	37.9 (36.0–41.1)	38.2 (35.6–40.5)*	38.1 (35.9–41.1)	38.0 (35.6–40.3)
Self-reported fever within the past 10 d lasting 2–7 d; currently <37.5	85 (7.1%)	66 (11.0%)	19 (3.2%)*	44 (5.9%)	41 (9.1%)^
Mild (37.5–38)	645 (53.8%)	315 (52.5%)	330 (55.0%)	397 (52.9%)	248 (55.1%)
Moderate (38.1–38.6)	240 (20.0%)	122 (20.3%)	118 (19.7%)	166 (22.1%)	74 (16.4%)
Severe (38.7–41.1)	230 (19.2%)	97 (16.2%)	133 (22.2%)	143 (19.1%)	87 (19.3%)
Received diagnosis for cause of fever	…	…	…	…	…
Yes	806 (67.2%)	233 (38.8%)	573 (95.5%)	497 (66.3%)	309 (68.7%)
No diagnosis	394 (32.8%)	367 (61.2%)	27 (4.5%)*	253 (33.8%)	141 (31.3%)
Hospital cause of fever diagnosis	…	…	…	…	…
Malaria	478 (39.8%)	215 (35.8%)	263 (43.8%)*	269 (35.9%)	209 (46.4%)**
Lassa fever	173 (14.4%)	1 (0.2%)	172 (28.7%)	125 (16.7%)	48 (10.7%)
Typhoid fever	10 (0.8%)	8 (1.3%)	2 (0.3%)	5 (0.7%)	5 (1.1%)
Other	36 (3%)	1 (0.2%)	35 (5.8%)	29 (3.9%)	7 (1.6%)
Other febrile illness	105 (8.8%)	7 (1.2%)	98 (16.3%)	68 (9.1%)	37 (8.2%)
Sickle Cell	3 (0.3%)	0 (0%)	3 (0.5%)	0 (0%)	3 (0.7%)
Disposition at discharge	…	…	…	…	…
Discharged to home	1125 (93.8%)	540 (90.0%)	585 (97.5%)*	684 (91.2%)	441 (98.0%)*
Discharged to an alternate care facility	5 (0.4%)	1 (0.2%)	4 (0.7%)	4 (0.5%)	1 (0.2%)
Deceased	48 (4.0%)	37 (6.2%)	11 (1.8%)	44 (5.9%)	4 (0.9%)
Left against medical advice	14 (1.2%)	14 (2.3%)	0 (0.0%)	10 (1.3%)	4 (0.9%)
Other	8 (0.7%)	8 (1.3%)	0 (0.0%)	8 (1.1%)	0 (0.0%)
Treatment	…	…	…	…	…
Antibiotics	385 (32.1%)	123 (20.5%)	262 (43.7%)*	255 (34.0%)	130 (28.9%)
Antifungal	1 (0.1%)	1 (0.2%)	0 (0.0%)	1 (0.1%)	0 (0.0%)
Anti-malarial	636 (53.0%)	340 (56.7%)	296 (49.3%)	350 (46.7%)	286 (63.6%)*
Antipyretic	146 (12.2%)	135 (22.5%)	11 (1.8%)*	83 (11.1%)	63 (14.0%)
Antiviral	174 (14.5%)	3 (0.5%)	171 (28.5%)*	128 (17.1%)	46 (10.2%)***
Anti-TB	2 (0.2%)	2 (0.3%)	0 (0.0%)	2 (0.3%)	0 (0.0%)
Self-treatment within the 72 h prior to seeking care					
Anti-malarial drug and an antibiotic	49 (4.1%)	4 (0.7%)	45 (7.5%)*	27 (3.6%)	22 (4.9%)*
Anti-malarial drug	567 (47.3%)	200 (33.3%)	367 (61.2%)*	340 (45.3%)	227 (50.4%)
Antibiotics	299 (24.9%)	137 (22.8%)	162 (27.0%)	217 (28.9%)	82 (18.2%)*
Symptoms experienced in the past 7 d	…	…	…	…	…
Headache	803 (66.9%)	393 (65.5%)	410 (68.3%)	493 (65.7%)	310 (68.9%)
Vomiting/nausea	400 (33.3%)	155 (25.8%)	245 (40.8%)*	218 (29.1%)	182 (40.4%)*
Abdominal pain	283 (23.6%)	79 (13.2%)	204 (34.0%)*	182 (24.3%)	101 (22.4%)
Cough	214 (17.8%)	151 (25.2%)	63 (10.5%)*	117 (15.6%)	97 (21.6%)***
Muscle pain	185 (15.4%)	97 (16.2%)	88 (14.7%)	144 (19.2%)	41 (9.1%)*
Arthralgia/joint pain	151 (12.6%)	82 (13.7%)	69 (11.5%)	113 (15.1%)	38 (8.4%)**
Diarrhea	140 (11.7%)	34 (5.7%)	106 (17.7%)*	90 (12.0%)	50 (11.1%)
Runny nose	102 (8.5%)	65 (10.8%)	37 (6.2%)	43 (5.7%)	59 (13.1%)*
Unusual/unexplained bleeding	47 (3.9%)	4 (0.7%)	43 (7.2%)*	36 (4.8%)	11 (2.4%)
Loss of appetite	34 (2.8%)	0 (0.0%)	34 (5.7%)*	17 (2.3%)	17 (3.8%)
Rash	22 (1.8%)	11 (1.8%)	11 (1.8%)	15 (2.0%)	7 (1.6%)
Jaundice	17 (1.4%)	3 (0.5%)	14 (2.3%)***	8 (1.1%)	9 (2.0%)
Dyspnea	13 (1.1%)	5 (0.8%)	8 (1.3%)	6 (0.8%)	7 (1.6%)
Weakness	11 (0.9%)	5 (0.8%)	6 (1.0%)	6 (0.8%)	5 (1.1%)
Chest pain	7 (0.6%)	2 (0.3%)	5 (0.8%)	3 (0.4%)	4 (0.9%)
Dizziness	6 (0.5%)	1 (0.2%)	5 (0.8%)	3 (0.4%)	3 (0.7%)
Seizure	4 (0.3%)	1 (0.2%)	3 (0.5%)	1 (0.1%)	3 (0.7%)
Coma	3 (0.3%)	0 (0.0%)	3 (0.5%)	2 (0.3%)	1 (0.2%)
Pinkeye	3 (0.3%)	0 (0.0%)	3 (0.5%)	2 (0.3%)	1 (0.2%)
Neck pain	2 (0.2%)	0 (0.0%)	2 (0.3%)	1 (0.1%)	1 (0.2%)
Health conditions (self-report)	…	…	…	…	…
High blood pressure	136 (11.3%)	76 (12.7%)	60 (10.0%)	135 (18.0%)	1 (0.2%)*
Diabetes	49 (4.1%)	39 (6.5%)	10 (1.7%)*	48 (6.4%)	1 (0.2%)*
Sickle Cell	25 (2.1%)	12 (2.0%)	13 (2.2%)	7 (0.9%)	18 (4.0%)**
HIV	22 (1.8%)	16 (2.7%)	6 (1.0%)^	19 (2.5%)	3 (0.7%)
Kidney issues	6 (0.5%)	3 (0.5%)	3 (0.5%)	6 (0.8%)	0 (0.0%)
Liver issues	6 (0.5%)	4 (0.7%)	2 (0.3%)	5 (0.7%)	1 (0.2%)
Heart issues	3 (0.3%)	2 (0.3%)	1 (0.2%)	3 (0.4%)	0 (0.0%)
Cancer	2 (0.2%)	1 (0.2%)	1 (0.2%)	2 (0.3%)	0 (0.0%)
Seizure disorders	2 (0.2%)	1 (0.2%)	1 (0.2%)	2 (0.3%)	0 (0.0%)
TB	1 (0.1%)	1 (0.2%)	0 (0.0%)	1 (0.1%)	0 (0.0%)
Other	21 (1.8%)	18 (3.0%)	3 (0.5%)	20 (2.7%)	1 (0.2%)***
None	963 (80.3%)	456 (76.0%)	507 (84.5%)**	538 (71.7%)	425 (94.4%)*

*P < .0001, **P < .001, ***P < .01, ^*P* < .05.

Abbreviations: FCT, Federal Capital Territory; TB, Tuberculosis.

A diagnosis from a hospital physician was received for 67.2% of participants, per data extracted from the patient chart, with significantly more recorded at Edo (95.5%, n = x) than FCT (38.8%). Malaria was the most common diagnosis from a hospital physician (39.8%, n = 478), followed by Lassa fever (14.4%, n = 173).

Most participants (93.8%) were discharged to home, but 4.0% died in the hospital. Death outcomes were higher among adults (5.9%) than children (0.9%), and higher in FCT (6.2%) than Edo (1.8%).

Febrile participants were most commonly treated with anti-malarial drugs (53%), antibiotics (32.1%), antivirals (14.5%), or antipyretics to reduce fever (12.2%). Antibiotics and antivirals were provided in Edo (43.7% and 28.5%) significantly more often than in FCT (20.5% and 0.3%). Many participants received multiple drugs.

## DISCUSSION

Of the 20 different pathogens the SAFIAN study detected, to our knowledge, this study is the first to report the molecular presence of eleven pathogens in humans in Nigeria. The pathogens were included in the SAFIAN study due to their potential for transmission to humans in Nigeria [18, 19]. Detection of such a sizable number of pathogens of public health significance, including hemorrhagic viruses [20], underscores the alarming fact that these cases could have gone undetected by hospitals, and undetected cases in the community or other parts of Nigeria are highly probable. From this group of largely vector-borne pathogens, several have been detected in Nigeria in animals or vectors [21] despite their non-detection in humans, emphasizing the ever-expanding impact of OneHealth pathogens and potential for spillover.

Although these research-use-only multiplex surveillance tools require confirmatory testing for diagnostic accuracy, these tools enable the potential for widespread detection to alert the public health communities of the presense of potential pathogens in their populations. Furthermore, by using TAC, SAFIAN was able to detect a potential AFI-causing pathogen in more than half the cases. Additionally, SAFIAN saw higher detection in children (62.9%). This supports other studies that have shown better pathogen detection in children compared with adults because of higher pathogen load [22].

Although not a replacement for confirmatory PCR, the findings from SAFIAN's serology for select pathogens further bolsters the TAC screening results for all three pathogens tested.

The widespread detection of *Rickettsia* spp. is significant, along with its role as a common coinfection in febrile illnesses in this population. Although human cases have not been identified in Nigeria, *Rickettsia* spp. has been identified in ticks from domesticated animals (cattle, camels) [23, 24] indicating potential for spillover. Limited studies have identified *Rickettsia* in humans in Africa; for example, an AFI study in Uganda identified Typhus group rickettsiosis (7.6%) and spotted fever group rickettsiosis (26.2%) [25–27]. Despite these findings, limited clinical signs such as rash or eschars and the inability to distinguish species-level pathogens hinder accurate diagnosis and appropriate treatment. These limitations highlight the urgent need for expanded surveillance, molecular speciation, and improved characterization of underdiagnosed rickettsial fevers in Nigeria.

Geographically, the SAFIAN study found few differences in the disease distribution between these two sites. The primary exception was Lassa fever, predominant only in Edo, but in nearly one-third of its AFI population. Other pathogens were identified in both locations or in such small numbers that differences were not significant. This critical finding is important for clinicians to understand these pathogens are widespread. The localization of Lassa fever in Edo is expected and is attributable to its specific vector, the multimammate rat (*Mastomys natalensis*) [28]. Notably, the presence of three cases in the other state suggests potential spread through alternative means.

Nigeria is a predominantly tropical country in West Africa, characterized by diverse geography and climates, ranging from arid to humid equatorial. While Edo is less developed than FCT, it also has more access to farms and wildlife. Our Edo study population included significantly more farmers and had the only participants reporting to be hunters. More participants in Edo's AFI population reported high-risk behaviors, including significantly fewer participants using bed nets and participants spending time in the forest/bush, putting them in contact with potential vectors such as rodents and insects (primarily mosquitos).

The study had a few limitations. The TAC assay included a target for Lassa virus; however, the assay did not reliably detect the lineages currently circulating in this population. The TAC assay designed by Thermo Fisher uses a bioinformatics approach that relies on sequences available in open-source databases. There was concern that the low detection of Lassa using TAC was likely due to targeting a specific lineage not commonly found in Nigeria. To resolve this issue, the study opted to include a single-pathogen PCR assay for Lassa virus and included results from the hospital-initiated PCR tests and additional testing conducted in our study [17]. Confirmatory testing was not feasible due to limited research funding. As a result, the *Plasmodium* spp. detection by TAC indicates the presence of parasite DNA but cannot confirm it as the cause of the current acute febrile episode.

The study recognizes the strengths and limitations of using a multi-pathogen screening tool such as TAC, which is for research use only. The TAC screening tool allows for a faster assessment of a large population to identify the potential pathogens likely to cause disease. This is informative when determining what other pathogens might be responsible for AFI besides malaria or if early detection is important for preventing spread of disease. However, the tool is limited and not diagnostic since the assays have not been validated to determine positive and negative predictive outcomes.

Based on the results, next steps should include confirmatory tesing, especially of pathogens of public health significance, and sequencing to determine rickettsial species.

## CONCLUSIONS

In conclusion, this epidemiologic study screening for the presence of 25 pathogens was instrumental in identifying pathogens of public health significance, including four never-before detected in Nigeria in humans, and seven additional pathogens never detected molecularly. These findings suggest that there are likely other undetected cases of these pathogens, highlighting the need for continued surveillance to monitor and respond to emerging health threats. Implementing clinical changes at the federal, state, and hospital levels to incorporate additional diagnostics and protocols for patients with potential AFI symptoms can enhance early detection and treatment, ultimately improving public health outcomes. The TAC results presented here are intended to serve as a screening tool to identify the presence of presumptive pathogen, which will guide and inform future studies involving clinical diagnostics and patient symptomatology.

The frequent misclassification of AFI as malaria, coupled with the lack of pathogen-specific diagnostics, hampers the ability of countries to detect outbreaks of emerging or re-emerging pathogens in their nascent stages. Effective AFI surveillance not only enables prompt outbreak detection but also supports the prioritization of public health interventions.

## Supplementary Material

ciaf500_Supplementary_Data

## Supplementary Data

Supplementary materials are available at *Clinical Infectious Diseases* online. Consisting of data provided by the authors to benefit the reader, the posted materials are not copyedited and are the sole responsibility of the authors, so questions or comments should be addressed to the corresponding author.
