# Supplementary material for: Descriptive Epidemiology of Acute Febrile Illness Patients in Nigeria: Pathogens of Global Public Health Significance Detected Using a Multi-Pathogen Detection Tool
Source: Clin Infect Dis. 2025 Nov 20;81(Suppl 4):S147–59. doi: 10.1093/cid/ciaf500 (PMC12631784; doi:10.1093/cid/ciaf500)
Supplement: ciaf500_Supplementary_Data [file ciaf500_supplementary_data.zip › Primary Outcomes_Supplemetary Material_2-June-2025.docx]

**Supplementary Material for SAFIAN Study**

**Sample Collection**

Consented participants provided whole blood and serum samples by venipuncture into ethylenediaminetetraacetic acid (EDTA) vacutainer (Becton Dickinson; Franklin Lakes, NJ) and serum vacutainer tubes (Becton Dickinson; Franklin Lakes, NJ) using aseptic techniques. The tubes were inverted to mix the contents with the blood. For serum samples, the tubes remained upright and undisturbed to allow for clotting and centrifuged to separate out the serum. Participants presenting with lesions characteristic to monkeypox provided a sample by swabbing the lesions following the recommended standard practice. The swabs were placed in a vial with viral transport media (Copan; Murrieta, CA). All samples were stored at -80ºC until ready for processing.

**Total Nucleic Acid (TNA) Extraction**

Whole blood and pox samples were initially treated with proteinase K (1 mg/mL) at 56ºC for 15 minutes. The treated samples were homogenized using the ZR BeadBashing Lysis tubes (Zymo; Irvine, CA) for 15 minutes and TNAs were extracted from the lysates using the Zymo Quick DNA/RNA Viral kit (Irvine, CA) following manufacturer’s instructions. The TNAs were eluted in 80 µL of nuclease-free water. For participants with whole blood and pox samples, 40 µL of elutions from each sample type were combined to provide a single 80 µL sample for polymerase chain reaction (PCR) analysis. All TNA samples were stored in the -80ºC until ready for TaqMan Array Card (TAC) PCR.

**TAC PCR**

For this study, we designed custom TaqMan Array Cards (TACs; ThermoFisher; Waltham, MA) using assays either commercially available (24 assays) or custom designed (2 assays) by the manufacturer (Table 1). The custom assays were designed using a proprietary algorithm which considers multiple criteria including melting temperature and nucleotide composition. The algorithm selects the best assays which have the highest specificity with target genomes and highest mismatch score for non-target genomes. The 18S rRNA gene was targeted as an internal control for determining TNA extraction efficiency and PCR inhibition. A custom positive template control was prepared by Thermo Fisher, containing a linearized plasmid pool of the 26 gene targets and nuclease-free water was used as a negative template control. The reactions were prepared by combining 55 µL of the TNA or control templates (positive control or negative control) with 50 µL of qScript XLT 1-Step RT-qPCR ToughMix (Quantabio; Beverly, MA). Ninety-five microliters of reaction mix were transferred to the appropriate sample reservoir and the TAC was prepared as instructed by the manufacturer.

**Table 1.** *List of Targets and Assays*

The layout of the assays on the TAC platform is shown in Figure 1 on the following page. Twenty-two of the 26 assays were run in duplicate. Four of the assays were run as singlets.

**Figure 1.**

*Layout of assays on the TAC platform*

**Table 1.**

*List of Targets and Assays*

| **Targets** | **Thermo Fisher Assay ID** |
| --- | --- |
| **Commercially available assays** |  |
| Zika virus (ZIKV) | APU64N6 |
| Chikungunya virus (CHIKV) | AP47XYP |
| O’nyong’nyong virus (ONNV) | AP327YV |
| Lassa virus (LASV) | APWC2PP |
| Crimean-Congo hemorrhagic fever virus (CCHFV) | APAAFNT |
| Hantavirus (HTNV) | APZTF69 |
| Rift Valley fever virus (RVFV) | APH6EX4 |
| Dengue virus (DENV) | APNKUKV |
| West Nile virus (WNV) | Vi04329496_s1 |
| Yellow fever virus (YFV) | APXGWAK |
| Hepatitis E virus (HEV) | APFVPF6 |
| *Trypanosoma brucei* (TBRUC) | APDJ3TY |
| *Leishmania* spp. (LEISH) | APGZKHF |
| *Bartonella* spp. (BART) | AP2XC9T |
| *Brucella* spp. (BRUC) | APCE6ZA |
| *Coxiella burnetii* (CBUR) | Ba06439618_s1 |
| *Leptospira* spp. (LEPT) | APEPXFN |
| Pan-*Salmonella* (pSALM) | Ba07921928_s1 |
| *Rickettsia* spp. (RICK) | APWCYF4 |
| *Yersinia pestis* (YPES) | APDJZNT |
| *Plasmodium* spp. (PLAS) | APKA7KX |
| *Neisseria meningitidis* (NMEN) | APNKRJZ |
| Monkeypox virus (MPOX) | Vi07922155_s1 |
| 18S rRNA (18S) – Control | Hs99999901_s1 |
| **Custom designed assays** |  |
| Marburg/Ebola viruses (MARV/EBOV) | APFVR2H |
| Pan-Orthopoxvirus (pOPX) | APAAH7N |
