# Supplementary figures and images for: Descriptive Epidemiology of Acute Febrile Illness Patients in Nigeria: Pathogens of Global Public Health Significance Detected Using a Multi-Pathogen Detection Tool
Source: Clin Infect Dis. 2025 Nov 20;81(Suppl 4):S147–59. doi: 10.1093/cid/ciaf500 (PMC12631784; doi:10.1093/cid/ciaf500)

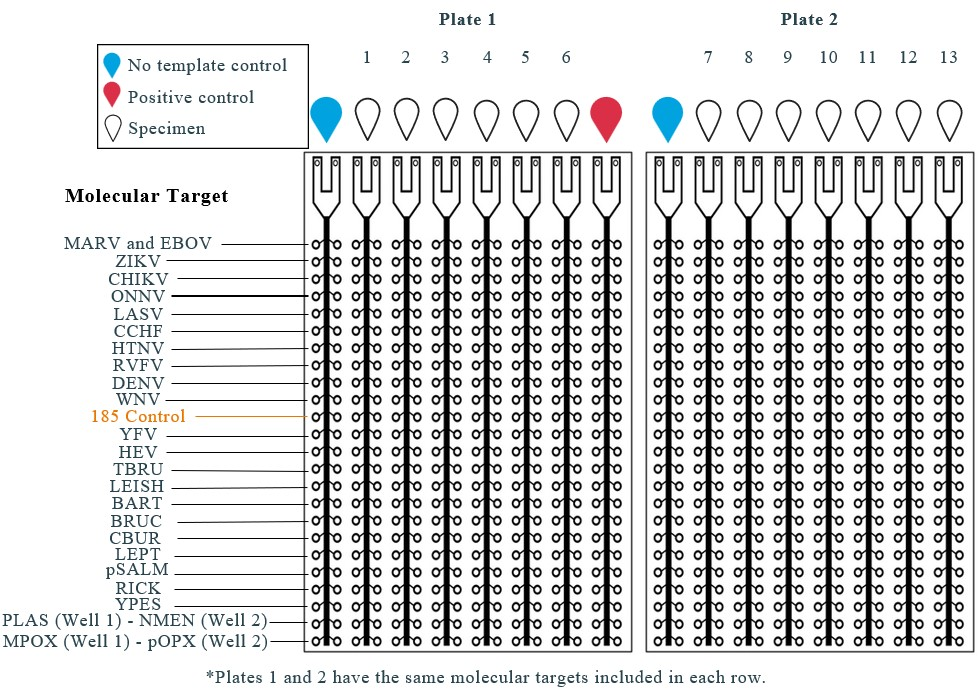

Supplement: ciaf500_Supplementary_Data [file ciaf500_supplementary_data.zip › Figure 1_Supplement Material.tif]
